# Singularities splitting phenomenon for the superposition of hybrid orders structured lights and the corresponding interference discrimination method

**DOI:** 10.1515/nanoph-2021-0814

**Published:** 2022-02-24

**Authors:** Baiwei Mao, Yange Liu, Wenzhe Chang, Liang Chen, Mao Feng, Huiyi Guo, Jiangyong He, Zhi Wang

**Affiliations:** Institute of Modern Optics, Tianjin Key Laboratory of Micro-scale Optical Information Science and Technology, Nankai University, Tianjin, 300350, China

**Keywords:** few-mode fibers, singularity, vortex array, vortex light

## Abstract

It is the basic characteristic of pure vortex light that there is a phase singularity at the origin. Such a singularity may be multiple degenerate, which determines the order of vortex light. Singularities splitting phenomenon means that singularities no longer concentrate at the origin but distribute around the space, usually occurring in impure vortex light. In this paper, we demonstrate the singularities splitting phenomenon and propose an analysis method, based on which one may rapidly estimate the modal components of impure vortex light. As two common singularity discrimination methods, the spiral and fork wire interference patterns are compared in distinguishing splitting singularities. The most widely used spiral interference pattern is revealed to be the worst form because of the low resolution. Instead, the fork wire interference pattern is with higher and easily adjusted resolution. 1‰ impurity is still able to be distinguished through fork wire interference patterns in the experiment.

## 1 Introduction

Vortex light is a kind of structured light characterized by the spiral wavefront and a central singularity. Because of its unique properties, vortex light is widely used in high-capacity communication [1, 2], quantum entanglement [3], optical tweezer [4, 5], and data storage [6, 7]. As the eigensolution of Helmholtz equation under cylindrical coordinates, vortex light has a series of azimuthal orders and radial orders, determining the different spatial distribution of amplitude and phase. For a nonzero azimuthal order vortex beam, there is a multidegenerate singularity located at the origin, where the intensity vanishes and the phase linearly changes around the origin.

Optical fiber is a natural container of vortex light. Depending on the refractive index distribution of fiber, several kinds of cylindrical functions may dominate the radial distribution of optical field in fiber, such as the Laguerre function for the square-index profile and Bessel function for the step-index profile. It’s a mutual property of cylindrical functions that a high-intensity area gradually moves away from the center along with the increasing azimuthal order. In other words, the central dark area is broader for the vortex light with higher azimuthal order.

To discriminate the singularity of vortex light, a Gaussian light with a different divergence or tilted angle is usually used to interfere with that. Under different divergence or tilted angles, the final interference image exhibits a spiral pattern or a fork wire pattern. During these two interference patterns, spiral interference patterns are more wildly used than fork wire interference patterns because of pretty. However, there exists a singularity splitting phenomenon that the singularities no longer concentrate at the center but distribute around the space if the structured light consists of hybrid order vortex lights, which may not be discriminated when using spiral interference patterns.

The singularities splitting phenomenon is common in multi-transverse mode laser [8], [9], [10], [11], which has been observed [12] as early before Allen et al. reveal the orbital angular momentum of light [13]. To arouse multiple transverse modes, the pump beam is usually obliquely incident into the laser crystal. As the emitted lights are formed by the coherent superposition of multiple transverse modes, the finally detected fields are messier than the pure transverse mode and usually exist several regular vortex phase areas around the space. As a result, researchers also call the complicated field ‘vortex array’. Besides multi-transverse mode laser, singularities splitting phenomenon also appears in few-mode fibers. In the past few years, with the development of fiber mode coupling devices, the generation of transverse modes in optical fiber gradually develops from the first-order mode [14], [15], [16], [17], [18], [19], [20] to the third-order mode [21], [22], [23], [24] or higher. As the increase of supported modes in fiber, the field becomes more and more complicated, which leads to some novel phenomena, including the singularities splitting phenomenon. Because fiber mode coupling devices like fiber grating and photonic lantern are sensitive to the status of the input beam, a little disturbance on fiber may cause the input beam to partially converted to unexpected order transverse modes. Researchers have generated the hybrid order vortex lights with splitting singularities deliberately [25] or not [26], [27], [28], but they have not focused on the splitting singularities. Also, their presenting spiral interference patterns are hard to discriminate these splitting singularities in their reported complicated optical fields.

In this paper, we demonstrate the singularities splitting phenomenon. In the beginning, the mathematical form of hybrid order vortex light that holds splitting singularities is discussed in theory. Through analysis, it’s revealed that the number and distribution of these singularities are related to the order, amplitude ratio, and phase difference of the combined vortex light. Meanwhile, these singularities perhaps possess different phase rotation directions. It’s common for an impure structured light that there are positive or negative singularities located at different places. Inversely, it’s possible to obtain the modal components according to the location and orientation of singularities. We discuss the relation between the modal components and the singularities in detail and summarize an analytical method. In this method, the main modal components of fields from fiber or multi-transverse mode laser can be estimated rapidly.

Furthermore, two kinds of interference patterns (spiral and fork wire) are discussed about the competence on discriminating the splitting singularities. The spiral interference pattern is found to be the worst method to distinguish the singularities because it possesses the lowest interference resolution among all the interference conditions. Instead, the fork wire pattern can easily distinguish these singularities and judge their rotation because of its high resolution. Using fork wire interference patterns, we experimentally discriminate all the splitting singularities of different hybrid order vortex lights from six-mode fiber, four-mode fiber, and from the spatial light modulator (SLM). 1‰ impurity is recognized by fork wire interference pattern in the experiment. Based on the location and orientation of these detected singularities, the modal components of the optical fields are recovered according to the proposed singularity analytical method, where the simulation results match well with that observed in the experiment.

## 2 Theory and simulation

Lights emitted from a few-mode fiber should consist of the eigenmodes that fiber can hold. As a result, the expression of the optical field in fiber is

(1) $$\begin{array}{l} E_{s}\left(r,\theta \right)=\Sigma_{l,m}A_{lm}e^{i\alpha_{lm}}\cdot F_{\left|l\right|m}\left(r\right){\text{e}}^{il\theta } \end{array},$$

where r and θ are the radius and azimuth related to the optics axis, l and m are the azimuthal and radial orders of structured lights, $A_{lm}$ and $\alpha_{lm}$ are the amplitude and phase of eigenmodes with the orders l and m , $F_{\left|l\right|m}\left(r\right)$ is the radial field function of structured light. Specially, $F_{01}\left(r\right)$ is the fundamental radial function, which can be regarded as Gaussian light.

**Figure 1:**
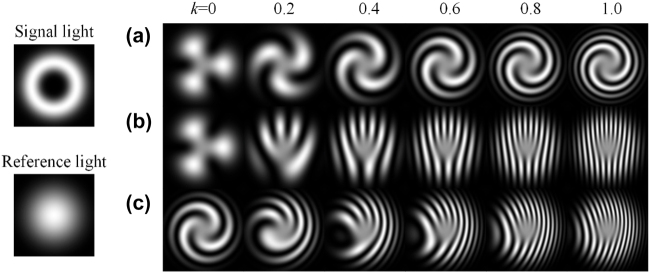
Interference patterns of a pure third azimuthal order vortex light when fixing (a) $k_{x}=0$ , (b) $k_{r}=0$ , (c) $k_{r}=0.6$ and scanning another k from 0 to 1.

It’s a common method to intuitively exhibit the azimuthal order of a vortex light by interfering with a fundamental mode with different divergence or tilted degree. Assuming z -direction as the optics axis, the expression is as below,

(2) $$\begin{array}{l} E_{i}=E_{s}{\text{e}}^{\text{i}k_{z}z}+A_{f}{\text{e}}^{\text{i}\alpha_{f}}\cdot F_{01}\left(r\right){\text{e}}^{\text{i}\left(k_{r}r^{2}+k_{x}x\right)}{\text{e}}^{\text{i}k_{z}z}, \end{array}$$

where $A_{f},\alpha_{f},F_{01}\left(r\right)$ are the amplitude, phase, and radial function of reference fundamental mode, $E_{s}$ and $E_{i}$ is the signal field before and after interference. $k_{x}$ is the transverse spatial frequency indicating the tilt degree, $k_{r}$ is proportional to radial spatial frequency indicating the spherical divergence degree. $F_{01}\left(r\right)$ is approximated to Gaussian function, different from $F_{lm}\left(r\right)$ with l>0 possessing a singularity at the center. The phase factor ${\text{e}}^{\text{i}k_{z}z}$ can be neglected as a common factor. Assuming m=1 at the beginning, for a pure single order vortex light, the complex amplitude $E_{i}$ and intensity $I_{i}$ after interfering with a fundamental mode are provided below

(3) $$\begin{array}{l} \left\{ \begin{array}{l} E_{i}=A_{l}F_{\left|l\right|1}\left(r\right){\text{e}}^{i\left(l\theta +\alpha_{l}\right)}+A_{f}F_{01}\left(r\right)e^{i\left(k_{r}r^{2}+k_{x}x+\alpha_{f}\right)}\\ I_{i}={\left[A_{l}F_{\left|l\right|1}\left(r\right)\right]}^{2}+{\left[A_{f}F_{01}\left(r\right)\right]}^{2}+2A_{l}A_{f}F_{\left|l\right|1}\left(r\right)F_{01}\left(r\right)\cos\left(l\theta -k_{r}r^{2}-k_{x}x+\alpha_{l}-\alpha_{f}\right) \end{array},\right. \end{array}$$

where the cosine term in the second row contributes to spiral or fork wire interference patterns. There are two factors $k_{r}$ and $k_{x}$ in the cosine term affecting the shape of interference patterns. When $k_{r}r^{2}+k_{x}x$ changes 2π , a bright stripe and a dark stripe appear and form a line pair. Line pairs can be considered as the sample of the singularity, where a more dense line pair can distinguish closer targets (see the Supplementary material). In other words, the density of line pairs is proportional to the interference resolution. In Figure 1, a pure third azimuthal order vortex light is chosen to be the signal beam and interfere with a Gaussian beam with different $k_{r}$ and $k_{x}$ . Figure 1(a) shows the patterns which fix $k_{x}=0$ and scanning $k_{r}$ from 0 to 1, corresponding to a reference beam with the same tilted degree and changing divergent degree. They are the classical spiral patterns. It should be emphasized that $k_{r}$ and $k_{x}$ in Figure 1 do not represent the concrete spatial frequency, but just indicate the trend of changing spatial frequency. Because the specific shape of the pattern also depends on the specific cylindrical function, it doesn’t make much sense to provide a concrete value of $k_{r}$ and $k_{x}$ . The selected values of $k_{r}$ and $k_{x}$ have been fitted to most of the experiment results shown in published articles [13], [14], [15], [16], [17], [18], [19], [20], [21], [22], [23], [24], [25], [26], [27], [28]. In this paper, the simulation radial function $F_{lm}\left(r\right)$ is the Laguerre–Gaussian function. Figure 1(b) shows the fork wires with $k_{r}=0$ and $k_{x}$ from 0 to 1, corresponding to an oblique incidence reference beam but the divergence is the same as the signal beam. For most cases, $k_{x}x$ change faster than $k_{r}r^{2}$ (see the supplementary material). That is why the number of line pairs in Figure 1(b) is more than that in Figure 1(a). Besides, Figure 1(c) shows the combined effects for $k_{r}=0.6$ and $k_{x}$ from 0 to 1, where the interference patterns are still the fork wire patterns if $k_{r}$ and $k_{x}$ exist simultaneously.

**Figure 2:**
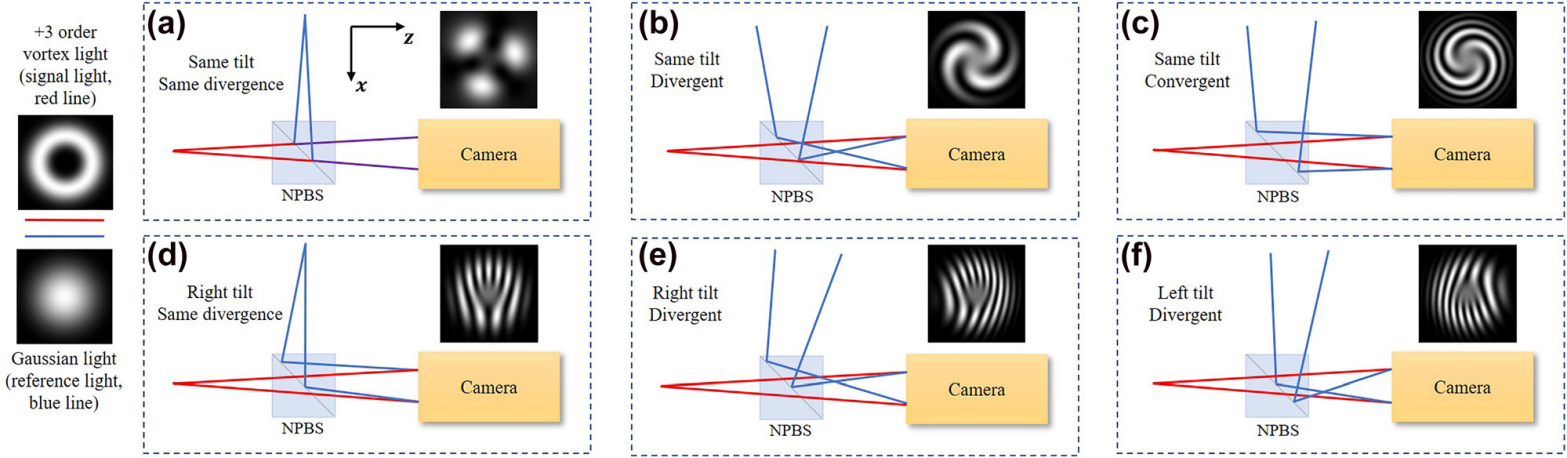
Diagram of the process that a pure third azimuthal order vortex light (red line) interferes with Gaussian lights (blue line) possessing (a) the same tilt and the same divergence (b) the same tilt and divergent (c) same tilt and convergent (d) right tilt and the same divergence (e) right tilt and divergent (f) right tilt and convergent relative to the signal vortex light. NPBS, nonpolarized beam splitter.

Figure 2 provides the concrete situations of interfering optics path where a pure third azimuthal order vortex light serves as the signal light (red line) and interferes with Gaussian beams (blue line) with different divergence and tilt degrees. As a reference, Figure 2(a) shows the Gaussian beam with the same divergence and the same tilt degree related to the signal vortex light. Compared with Figure 2(a), the spiral interference patterns in Figure 2(b) and (c) are caused by the relative divergence between these two beams. Further comparing Figure 2(b) with Figure 2(c), with the same spot size of the reference beam, the stripe is finer when the signal beam and the reference beam possess opposite divergence (Figure 2(c)). Under this condition, a positive singularity should correspond to the clockwise spiral pattern, while a negative singularity corresponds to the counter-clockwise spiral pattern. However, researchers are used to relating the counter-clockwise spiral pattern to a positive singularity, which has a thick interference stripe compared with that in the other condition. Even though it is not good, to cater to the habits of most people, we still link the counter-clockwise spiral pattern to a positive singularity in the rest of this paper. Comparing Figure 2(a) with Figure 2(d), relative tilt degree causes the fork-wire interference pattern. Similarly, the right tilt reference beam leads to the upper open fork pattern for positive vortex signal light while the left tilt reference beam leads to the downer open one if with little divergence between the signal light and the reference light.

Researchers are used to presenting the spiral interference patterns because of the pretty [13], [14], [15], [16], [17], [18], [19], [20], [21], [22], [23], [24], [25], [26], [27], [28]. However, compared with fork wire, spiral patterns are with the lowest resolution. As known, interference resolution depends on the spatial frequency difference between signal and reference beams. Although it’s possible to improve the resolution by enlarging the factor $\left|k_{r}\right|$ , but the enlargement of | $k_{r}$ | will cause the change of the light spot, leading to the mismatch of spot size of two interfering beams. On the other side, the change of $k_{x}$ do not change the spot size, and therefore avoid this problem. For a single order structured light, the increase of interference resolution has little effect, because all the singularities degenerate at the center. For hybrid-order structured lights, the interference resolution will play an important role. Before discussing the interference patterns of hybrid-order structured light, the singularities splitting phenomenon of hybrid-order vortex light should be introduced first.

### 2.1 Singularities splitting phenomenon of hybrid-order vortex light

The singularities splitting phenomenon means that there exist several singularities besides the one located at the origin. It is a common phenomenon for structured lights consisting of different orders vortex lights. These non-origin singularities are usually close to each other and may also have positive or negative topological charges. As a result, it has higher requirements for interference method to distinguish all singularities of hybrid-order vortex light, different from those pure vortex lights only with a single multidegenerated singularity at the origin.

Take a little more complicated case for an example, structured light combined by two different orders vortex lights $V_{l_{1}m_{1}}$ and $V_{l_{2}m_{2}}$ , the complex amplitude of such an arbitrary beam is

(4) $$\begin{array}{l} E_{s}\left(r,\theta \right)=V_{l_{1}m_{1}}+V_{l_{2}m_{2}}=A_{1}F_{\left|l_{1}\right|m_{1}}\left(r\right){\text{e}}^{\text{i}\left(l_{1}\theta +\alpha_{l_{1}}\right)}+A_{2}F_{\left|l_{2}\right|m_{2}}\left(r\right){\text{e}}^{\text{i}\left(l_{2}\theta +\alpha_{l_{2}}\right)} \end{array}.$$

**Figure 3:**
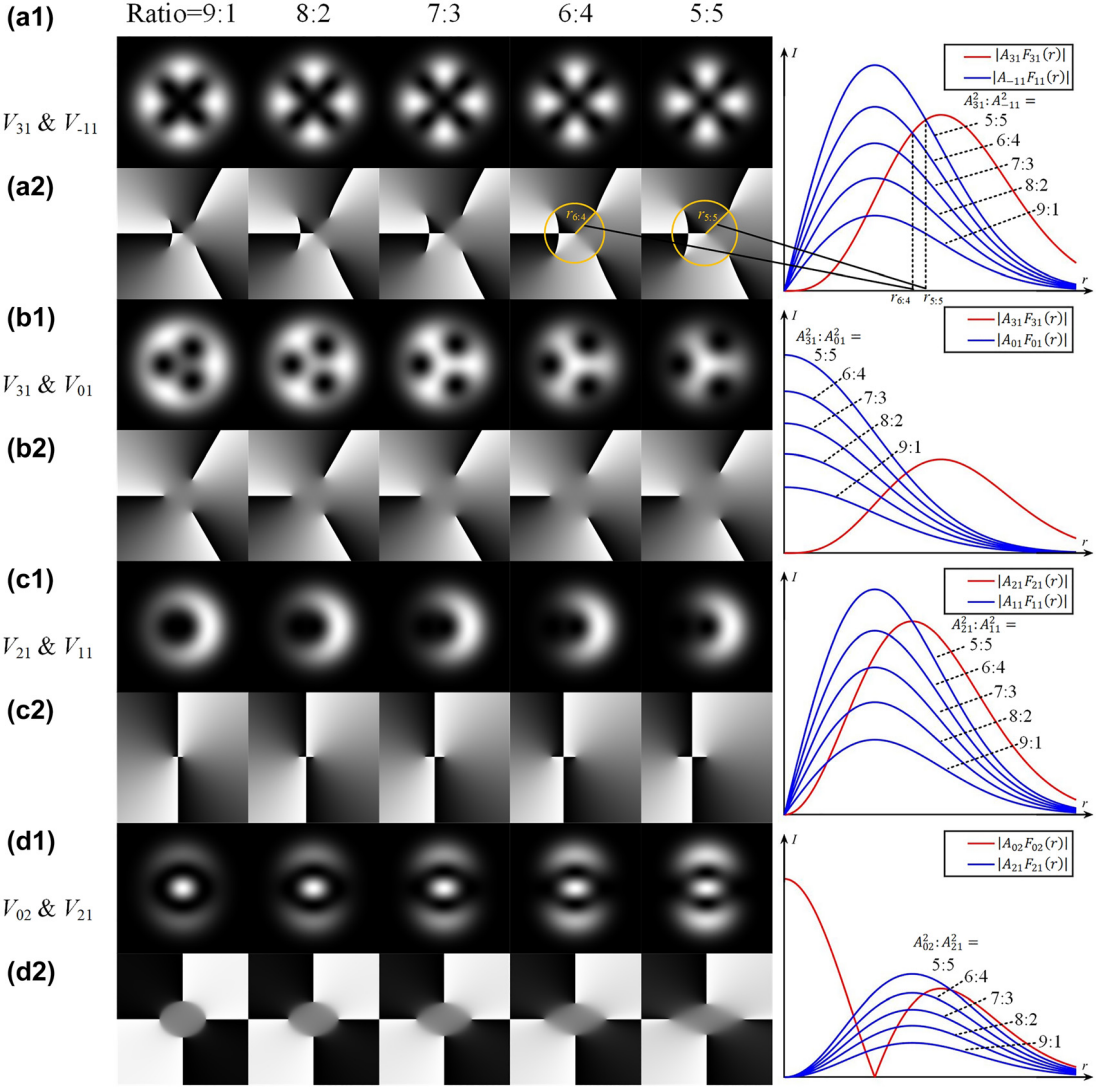
Singularities splitting phenomenon of hybrid-order vortex lights. The intensity and phase patterns of structured light combined by ((a1) and (a2)) $V_{31}$ and $V_{-11}$ , ((b1) and (b2)) $V_{31}$ and $V_{01}$ , ((c1) and (c2)) $V_{21}$ and $V_{11}$ , ((d1) and (d2)) $V_{02}$ and $V_{21}$ vortex lights with the intensity ratio from 9:1 decreasing to 5:5. The right curves are the corresponding radial functions along with the change of the total intensity ratio.

For convenience, $l_{1}>l_{2}$ is assumed. Because the combined lights are continuous in the space, the areas that phases change sharply are only possible to appear around the point where the two beams destructively interfere. The polar coordinates (r,θ) of the singularities should satisfy

(5) $$\begin{array}{l} \begin{array}{l} \left|A_{1}F_{\left|l_{1}\right|m_{1}}\left(r\right)\right|=\left|A_{2}F_{\left|l_{2}\right|m_{2}}\left(r\right)\right|\\ \left(l_{1}-l_{2}\right)\theta +\alpha_{l_{1}}-\alpha_{l_{2}}=\left\{ \begin{array}{l} \left(2n+1\right)\pi ,F_{\left|l_{1}\right|m_{1}}\left(r\right)\cdot F_{\left|l_{2}\right|m_{2}}\left(r\right)>0\\ 2n\pi ,F_{\left|l_{1}\right|m_{1}}\left(r\right)\cdot F_{\left|l_{2}\right|m_{2}}\left(r\right)<0 \end{array}\right. \end{array} \end{array},$$

where n∈Z . For the first equation, it’s obvious that the origin (0,0) is one of the roots if $l_{1}>l_{2}\neq 0$ because the higher-order cylindrical function vanishes at the origin. Meanwhile, because the lower cylindrical function $F_{\left|l\right|m}\left(r\right)$ is much bigger than that with adjacent higher azimuthal order, the degenerate order of the singularity at the origin is determined by the lower-order vortex light, even for trace lower-order vortex light. As shown in Figure 3(a1), (a2), (c1) and (c2), even the intensity ratio between two different orders beams comes to 9:1, the order of central singularity is still dominated by the lower order vortex light. As for the second equation about azimuthal angle θ in Eq. (5), $l_{1}-l_{2}$ roots are located around the circle if $F_{\left|l_{1}\right|m_{1}}\left(r\right)\cdot F_{\left|l_{2}\right|m_{2}}\left(r\right)>0$ or $F_{\left|l_{1}\right|m_{1}}\left(r\right)\cdot F_{\left|l_{2}\right|m_{2}}\left(r\right)<0$ holds in the whole space. All the vortex lights with $m_{1}=m_{2}=1$ meet this condition. For example, the number is 3 minus −1 equal to 4 for Figure 3(a) and is 2 minus 1 equal to 1 for Figure 3(c). Meanwhile, the radius of non-origin singularities indicates the intensity ratio of the combined vortex lights. As shown in Figure 3, the right curves provide the trends of the intensities $I_{1}={\left|A_{1}F_{\left|l_{1}\right|m_{1}}\left(r\right)\right|}^{2}$ and $I_{2}={\left|A_{2}F_{\left|l_{1}\right|m_{2}}\left(r\right)\right|}^{2}$ changing along with the radius r . Because $F_{lm}\left(r\right)$ has been normalized that $2\pi \int {\left|F_{lm}\left(r\right)\right|}^{2}dr=1$ , the total intensity ratio is equal to $A_{1}^{2}:A_{2}^{2}$ . To satisfy the first equation of Eq. (5), the radius of the singularities should be located at the intersection points of the curves painted two colors. It is indicated that the radii of non-origin singularities increase along with the growth of the intensity of lower-order vortex lights.

Based on the second equation of Eq. (5), the concrete location of θ is also associated with the phase difference $\alpha_{l_{1}}-\alpha_{l_{2}}$ . However, this factor does not affect the shape of the interference pattern and just contributes to an unimportant rotation. For this reason, $\alpha_{l_{1}}-\alpha_{l_{2}}$ defaults to 0 in Figure 3.

For the case that m≠1 , it’s needed to divide the space as different rings. Some of them satisfy $F_{\left|l_{1}\right|m_{1}}\left(r\right)\cdot F_{\left|l_{2}\right|m_{2}}\left(r\right)>0$ while the others satisfy $F_{\left|l_{1}\right|m_{1}}\left(r\right)\cdot F_{\left|l_{2}\right|m_{2}}\left(r\right)<0$ . As shown in Figure 3(d1) and (d2), the structured lights combined by $V_{02}$ and $V_{21}$ , the radius dividing these two rings is chosen according to the zero point of $F_{02}\left(r\right)$ . For the inner ring, as the analysis above, there appear two singularities at θ=±π/2 . Back to the second equation of Eq. (5), the analysis of the outer ring is similar to that of the inner ring. There should also be two singularities in the outer ring, but with an extra rotation of $\Delta \theta =\pi /\left(l_{1}-l_{2}\right)=\pi /2$ . Meanwhile, as shown in the curves of $\left|A_{02}F_{02}\left(r\right)\right|$ and $\left|A_{21}F_{21}\left(r\right)\right|$ , the dominated light is a sandwich structure as $V_{02},V_{21}$ and $V_{02}$ . Thus, the singularities orientation of the outer ring should be inverse relative to that of the inner ring.

**Figure 4:**
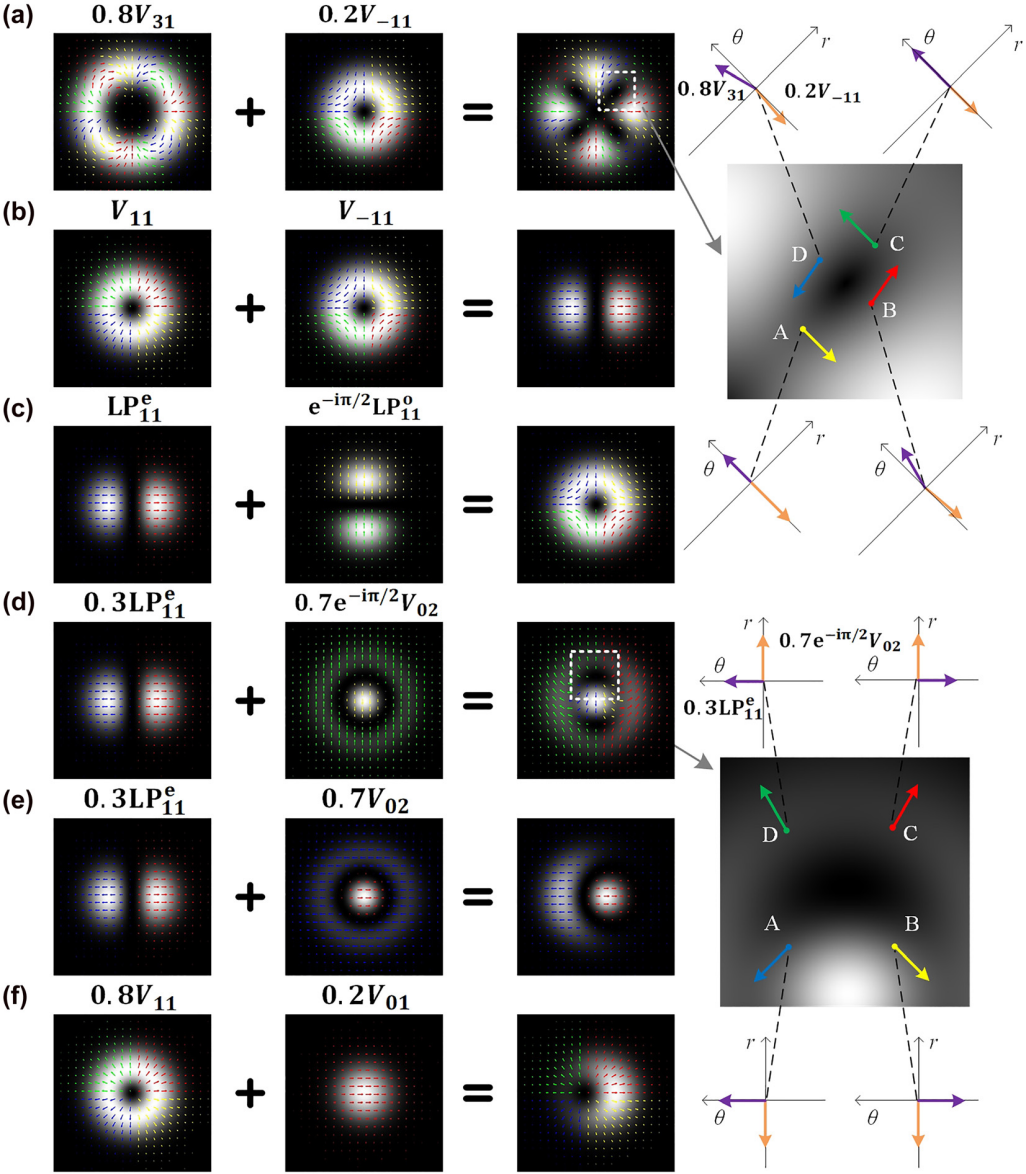
Superimposed patterns of two structured lights with auxiliary arrows indicating complex vectors. Structured lights consist of (a) $0.8V_{31}$ and $0.2V_{-11}$ , (b) $V_{11}$ and $V_{-11}$ , (c) ${\text{LP}}_{11}^{\text{e}}$ and ${\text{e}}^{-\text{i}\pi /2}{\text{LP}}_{11}^{\text{o}}$ , (d) $0.3{\text{LP}}_{11}^{\text{e}}$ and $0.7{\text{e}}^{-\text{i}\pi /2}V_{02}$ , (e) $0.3{\text{LP}}_{11}^{\text{e}}$ and $0.7V_{02}$ , (f) $0.8V_{11}$ and $0.2V_{01}$ . The right graphs are the enlarged images framed by the white box of (a) and (d).

As the superposition of two hybrid vortex lights may generate non-origin singularities, an obvious question is, why the combination of +1 azimuthal order vortex light and −1 azimuthal order vortex light does not generate two non-origin singularities, but form the linearly polarized (LP) mode without any singularity when $A_{1}=A_{2}$ , as shown in Figure 4(b)? To solve the problem, it’s helpful to survey the superposition of hybrid-order vortex lights under the complex vector sight. As shown in Figure 4, the two terms on the right side of Eq. (4) are exhibited as the complex vectors changing with spatial coordinates. The length of the complex vector is proportional to the amplitude while the orientation of that is equal to the phase at each point. In Figure 4, the phases are divided according to four quadrants and the corresponding arrows are painted in different colors, where red, green, blue, and yellow colors represent the first to the fourth quadrants respectively. As a result, the point surrounded by arrows of four colors is the singularities. Let’s firstly discuss Figure 4(a), where the field consists of $0.8V_{31}$ and $0.2V_{-11}$ . Arrows painted four colors surround those areas around the destructive points. That means, the phase only changes sharply in these areas. Assume the destructive points as $\left(r_{d},\theta_{d}\right)$ , its surrounded points as $A=\left(r_{d}-\delta r,\theta_{d}\right)$ , $B=\left(r_{d},\theta_{d}-\delta \theta \right)$ , $C=\left(r_{d}+\delta r,\theta_{d}\right)$ and $D=\left(r_{d},\theta_{d}+\delta \theta \right)$ . Take the singularity whose $\theta_{d}=\pi /4$ as an example (framed by a white box in Figure 4(a)), the amplitudes of $0.8V_{31}$ and $0.2V_{-11}$ are equal, while the phase of $V_{31}$ is $3\pi /4\frac{1}{2}$ and the phase of $V_{-11}$ is −π/4 . Therefore, at the destructive point, a 3π/4 purple arrow and a −π/4 orange arrow with the same amplitude destructively interfere. For point A, the phase of both $V_{31}$ and $V_{-11}$ are the same as that at the destructive point, while the amplitude of $V_{-11}$ is larger than $V_{31}$ . For point B, the amplitude of both $V_{31}$ and $V_{11}$ are the same, while $V_{31}$ rotates a relatively larger angle than that of $V_{-11}$ . The analyses of points C and D are similar. Based on the analyses, we may draw the synthetic arrows at these points. It is found that the complex vector rotates around the destructive point, which exhibits phase vortex property. Indeed, it’s the differential approximation of derivatives. Using the condition Eq. (5), the derivatives of the destructive point can be derived as

$$\begin{array}{l} \frac{\partial E_{\text{s}}}{\partial r}{|}_{r=r_{d},\theta =\theta_{d}}=\frac{\partial F_{\left|l_{1}\right|m_{1}}\left(r_{d}\right)}{\partial r}A_{1}{\text{e}}^{\text{i}\left(l_{1}\theta_{d}+\alpha_{l_{1}}\right)}+\frac{\partial F_{\left|l_{2}\right|m_{2}}\left(r_{d}\right)}{\partial r}A_{2}{\text{e}}^{\text{i}\left(l_{2}\theta_{d}+\alpha_{l_{2}}\right)} \end{array}$$

(6) $$\begin{array}{l} \approx A_{1}\frac{\partial F_{\left|l_{1}\right|m_{1}}\left(r_{d}\right)}{\partial r}\mp A_{2}\frac{\partial F_{\left|l_{2}\right|m_{2}}\left(r_{d}\right)}{\partial r}\neq 0 \end{array},$$

$$\frac{\partial E_{\text{s}}}{\partial \theta }{|}_{r=r_{d},\theta =\theta_{d}}=\text{i}A_{1}l_{1}F_{\left|l_{1}\right|m_{1}}\left(r_{d}\right){\text{e}}^{\text{i}\left(l_{1}\theta_{d}+\alpha_{l_{1}}\right)}+\text{i}A_{2}l_{2}F_{\left|l_{2}\right|m_{2}}\left(r_{d}\right){\text{e}}^{\text{i}\left(l_{2}\theta_{d}+\alpha_{l_{2}}\right)}\approx l_{1}-l_{2}\neq 0$$

where the sign of the first equation in Eq. (6) depends on the condition of the second equation in Eq. (5), which is negative when $F_{\left|l_{1}\right|m_{1}}\left(r_{d}\right)\cdot F_{\left|l_{2}\right|m_{2}}\left(r_{d}\right)>0$ and positive when $F_{\left|l_{1}\right|m_{1}}\left(r_{d}\right)\cdot F_{\left|l_{2}\right|m_{2}}\left(r_{d}\right)<0$ . The azimuthal derivative at the destructive point indicates that a field consisting of any two different order vortex lights may possess non-origin singularities. However, it should simultaneously satisfy the first equation of Eq. (6). A counterexample is the superposition of $V_{11}$ and $V_{-11}$ with the same amplitude $A_{1}=A_{2}$ , and they share the same radial function $F_{\left|l_{1}\right|m_{1}}\left(r\right)=F_{\left|l_{2}\right|m_{2}}\left(r\right)=F_{11}\left(r\right)$ . It’s not hard to see the radial derivative is zero. Thus, it’s not able to form any singularity but form the LP mode. Intuitively, Figure 4(b) only possesses the arrows painted two colors, compared with the singularities surrounded by arrows painted four colors in other graphs.

Back to the discussion just now, a classical superposition is to generate vortex light by combining an LP even mode ${\text{LP}}_{lm}^{\text{e}}$ and an LP odd mode ${\text{LP}}_{lm}^{\text{o}}$ with π/2 phase difference, as shown in Figure 4(c). The fundament of the principle is to provide arrows in the four quadrants surrounding the origin. Similarly, if one wants to form non-origin singularities based on ${\text{LP}}_{11}$ , the arrows of the other two quadrants should be provided, where $V_{02}$ with π/2 phase difference just satisfy this condition, shown in Figure 4(d). At the same time, the upper singularity is positive while the lower singularity is negative because the $V_{02}$ provided arrows are symmetrical about the *x*-axis. As a comparison, the combination of ${\text{LP}}_{11}^{\text{e}}$ and $V_{02}$ with the same phase difference is shown in Figure 4(e), which is not able to form singularities because they both do not provide green and yellow arrows at each point.

Considering the upper singularity framed by a white box in Figure 4(d), it’s clear for the arrows at points $\left(r_{d}-\delta r,\theta_{d}\right)$ , $\left(r_{d},\theta_{d}-\delta \theta \right),\left(r_{d}+\delta r,\theta_{d}\right)$ and $\left(r_{d},\theta_{d}+\delta \theta \right)$ , which should be down, left, up, and right. Additionally, the arrows at the points $A=\left(r_{d}-\delta r,\theta_{d}+\delta \theta \right)$ , $B=\left(r_{d}-\delta r,\theta_{d}-\delta \theta \right),C=\left(r_{d}+\delta r,\theta_{d}-\delta \theta \right)$ and $D=\left(r_{d}+\delta r,\theta_{d}+\delta \theta \right)$ are discussed. Even the phase changes periodically along the circle near the singularity, this change is not steady as a pure vortex light, which is with totally equal amplitude and linearly changing phase on the circle around the original singularity. In Figure 4(d), it’s different about the degree of change at point A, B, and that at point C, D.

At last, the structure light consisting of $V_{11}$ and $V_{01}$ is provided in Figure 4(f) to introduce the influence of a fundamental mode on an existing first-order singularity. A phase uniform region (like $V_{01}$ , one of the rings of $V_{0m}$ and one of the petals of ${\text{LP}}_{l1}$ ) may translate the former singularity along a specific direction according to the concrete phase difference between the singularity and the phase uniform region. In Figure 4(f), the phase of the fundamental mode is 0. It will constructively interfere with the right part of $V_{11}$ and destructively interfere with the left part of $V_{11}$ so that the singularity is translated to the left. In a word, the former singularity will move along the direction opposite to the arrows of the phase uniform area. The function of the fundamental mode to translated an existed singularity is common in the reported results in two-mode fiber [16], [17], [18], [19], [20, 29], [30], [31], [32], [33], [34], [35], [36], but people usually regard their reported results as the pure mode so that neglect the effect of the fundamental mode. This property will be used to explain the experimental results in the following section.

It should be mentioned that Eq. (6) is not able to judge even order non-origin singularity, which may possess the same complex vectors on the opposite side of r or θ directions at the destructive point. The most rigorous method is to calculate the azimuthal derivative based on the coordinate center located at the destructive point. However, this method leads to a long formula that is hard to exhibit and use. We have not observed degenerate non-origin singularity with more than one order in fiber systems and previously reported articles about multimode laser. In other words, the order of non-origin singularity can default to 1, which means the phase will not sharply change more than 2π around the non-origin destructive point. Therefore, Eq. (6) or the arrows judgment method are enough and convenient to be used.

### 2.2 Comparison between spiral and fork wire interference patterns

**Figure 5:**
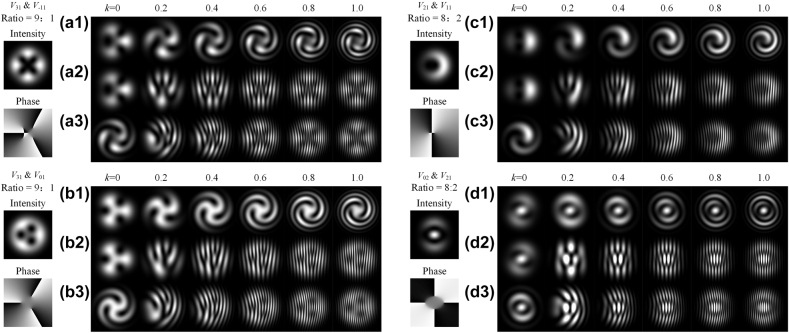
Interference patterns of structured lights consisting of (a) $V_{31}$ and $V_{-11}$ with intensity ratio 9:1, (b) $V_{31}$ and $V_{01}$ with intensity ratio 9:1, (c) $V_{21}$ and $V_{11}$ with intensity ratio 8:2, and (d) $V_{02}$ and $V_{21}$ with intensity ratio 8:2, when fixing ((a1), (b1), (c1) and (d1)) $k_{x}=0$ , ((a2), (b2), (c2) and (d2)) $k_{r}=0$ , ((a3), (b3), (c3) and (d3)) $k_{r}=0.4$ and scanning the other k from 0 to 1.

Singularities splitting phenomenon has been introduced above. Different from pure vortex light with a single order, impure vortex lights have several non-origin singularities appearing beside the one located at the origin. As some close targets need to be distinguished, the resolution of the interference method should be required. However, the conventional spiral interference pattern may not be competent here. Back to Eq. (3), the interference condition can be characterized by the factors $k_{r}$ and $k_{x}$ . They represent the difference of divergence and tilt degree between the signal light and reference light, respectively, as also mentioned in Figure 2. As shown in Figure 5(a1), even in the largest $k_{r}$ , the spiral interference patterns still reveal little information about the singularities. As a comparison, in Figure 5(a2) and (a3), when imposing a suitable tilted degree $k_{x}$ , all the singularities can be distinguished well. Concretely, a lower opening fork wire is at the center and four upper opening fork wires are located around, indicating a negative singularity at the center and four positive singularities located around. Further comparing Figure 5(a2) with Figure 5(a3), the only difference is $k_{r}=0$ in Figure 5(a2) and $k_{r}=0.4$ in Figure 5(a3). It’s found that the divergence degree $k_{r}$ affects little about the judgment of these singularities. As a result, it’s recommended to adjust the spot size by adjusting $k_{r}$ and choose appropriate resolution by tilting the incident angle of the reference beam.

On the other side, if m=1 , under the same intensity ratio, the radii of non-origin singularities are smaller for the combination of vortex lights with closer absolute azimuthal orders, so that need a higher interference resolution. Comparing Figure 5(a3) with Figure 5(c3), the absolute value difference of the two combined azimuthal orders are 3−1=2 and 2−1=1 . Therefore, the radial functions $F_{\left|l_{1}\right|m_{1}}\left(r\right)$ and $F_{\left|l_{2}\right|m_{2}}\left(r\right)$ are closer for Figure 5(c3), leading to the smaller radii of the splitting singularities under the same intensity ratio. As shown, the singularities are distinguished roughly when $k_{x}=0.6$ in Figure 5(a3), but $k_{x}=0.8$ in Figure 5(c3). The singularities will be closer in Figure 5(c3) if changing the ratio from 8:2 to 9:1 and verifying the conclusion better.

For the case combined with structured lights with different radial orders, the spiral interference patterns still reveal nearly no information from the combination of $V_{02}$ and $V_{21}$ in Figure 5(d1), but the two pairs of singularities with opposite orientations are clearly discriminated by fork wire interference patterns shown in Figure 5(d2) and (d3). It should be mentioned that Figure 5(d2) and (d3) are overexposed to make the singularities clear, due to the large contrast of this combination.

## 3 Experiment results

**Figure 6:**
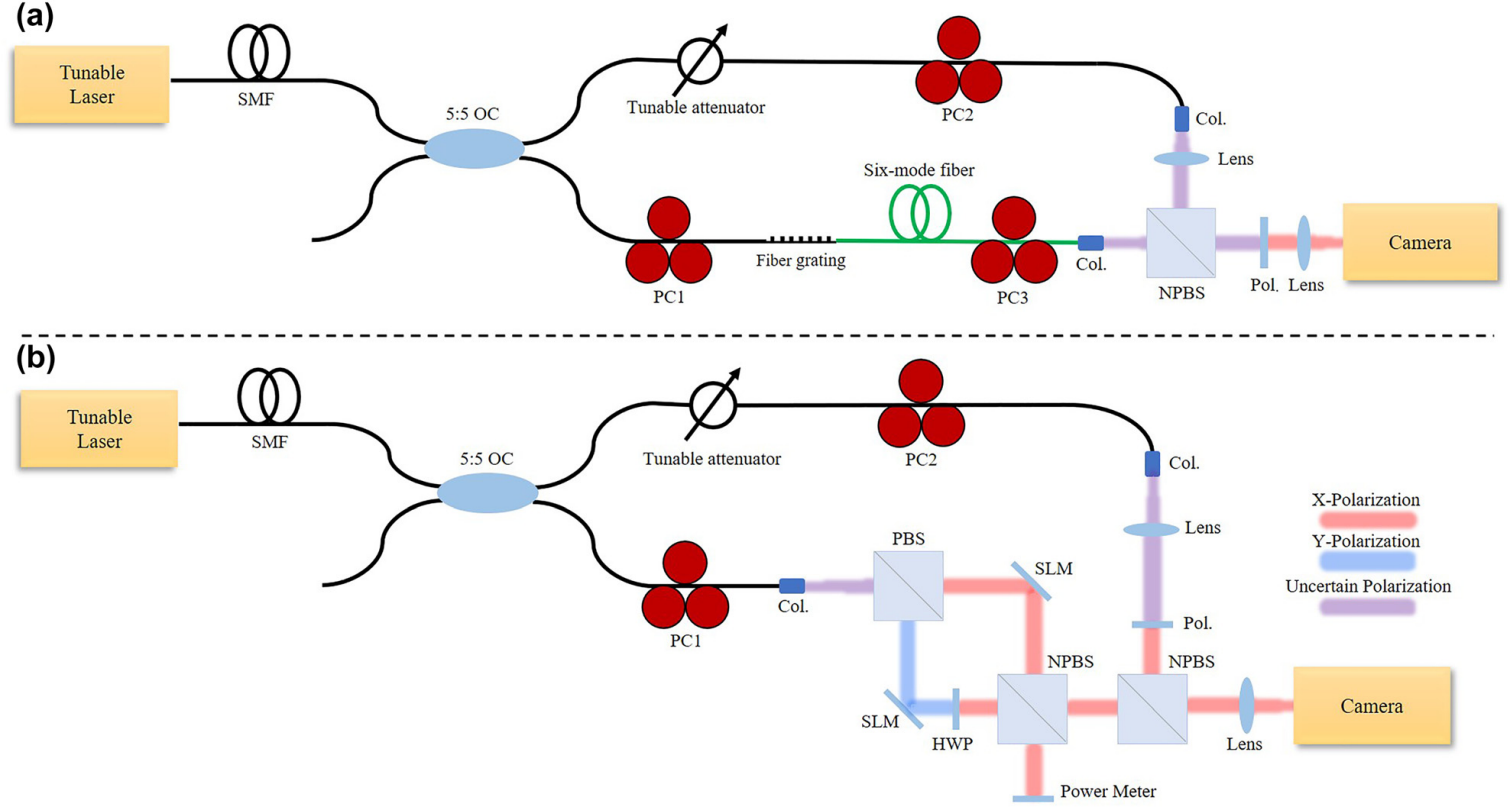
Experiment setup of Mach Zehnder interference system to recognize splitting singularities in which signal beam is generated by (a) fiber system and (b) spatial system. SMF, single-mode fiber; OC, optics coupler; PC, polarization controller; Col, collimator; PBS, polarized beam splitter; NPBS, non-polarized beam splitter; HWP, half-wave plate; SLM, spatial light modulator; Pol., polarizer.

Two experiment setups are established to verify the singularities splitting phenomenon and compare the practical performance between spiral and fork wire interference patterns. The most essential motivation is to estimate the components of the optical fields from the few-mode fiber. Thus, the generation method of the first experimental setup is based on fiber mode coupler devices and a six-mode fiber, as shown in Figure 6(a). However, it’s hard to precisely adjust the field in fiber. Therefore, another experiment is designed. The generation of hybrid-order vortex modes is based on two SLMs, by which the intensities and phases of combined vortex lights can be quantitatively controlled.

The fiber system shown in Figure 6(a) is introduced firstly. Beginning from the top-left fiber part, a tunable laser (KEYSIGHT 81600B, 1460 nm–1640 nm) is followed by a single-mode fiber (SMF) connected to a 5:5 optical coupler (OC) that divides light into two paths. The lower path is the signal path, to generate impure hybrid orders vortex light. The emitted light firstly passes a polarization controller (PC1) to adjust the polarization. Then, two long-period fiber gratings (LPFGs), −15 dB at 1556.4 nm for $V_{31}$ and −20 dB at 1551.1 nm for $V_{21}$ , are respectively set to change the input fundamental mode to specific higher-order modes. Though LPFGs can realize high transformation efficiency at the resonance wavelength, we do not set the wavelength as the resonance wavelength, but stagger a little from that to arouse vortex modes with other orders besides the desired order. After the LPFG, PC3 is set to adjust the amplitudes and phases of the excited higher-order modes to generate different modal combinations.

The upper path is the reference path, to provide fundamental mode with a suitable intensity, which is adjusted by a tunable attenuator. As for the spatial part, that is a classical Mach Zehnder interference system. Both signal light and reference light firstly pass through a collimator, respectively. The signal light is collimated, while the reference light is adjusted to match the spot size of the signal light and usually remains a little divergence through an extra lens. These two beams concentrate after a nonpolarized beam splitter (NPBS) and finally pass through a polarizer before falling on the camera (FIND-R-SCOPE 85706, 400–1800 nm). The lens before the camera is used to image, while PC2 and the polarizer are used to adjust the intensity of the reference beam.

For the spatial generation experimental setup shown in Figure 6(b), the beginning fiber part is the same. Then, light from the lower path (SMF, fundamental mode) passes through a polarized beam splitter (PBS), and respectively modulated by two SLMs (Holoeye Pluto 2.1) and converted into different orders pure vortex lights. Here, PC1 is used to adjust the intensity ratio of two vortex lights. A half-wave plate (HWP) whose fast axis at $45^{\circ }$ is placed in the *y* polarization beam path to change *y* polarization to *x* polarization. It should be mentioned that the $45^{\circ }$ placed SLMs do not represent the real setup, where several reflected mirrors are neglected. Then, these two vortex lights with different orders, the same divergence, and the same inclination angle interfere with each other via a 5:5 NPBS. One branch after the NPBS performs as the signal beam while the other branch is used to measure the power of each vortex light respectively. The other parts are the same as Figure 6(a).

**Figure 7:**
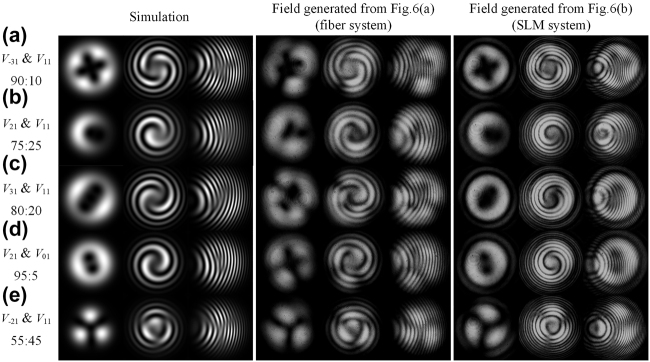
Experiment and simulation results of hybrid-order vortex lights generated by fiber system and SLM system. The signal structured lights mainly consist of (a) $V_{-31}$ and $V_{11}$ , (b) $V_{21}$ and $V_{11}$ , (c) $V_{31}$ and $V_{11}$ , (d) $V_{21}$ and $V_{01}$ , (e) $V_{-21}$ and $V_{11}$ vortex lights. The ratios below the modal combination are the simulated intensity ratio of the two combined vortex lights.

Figure 7 provides the experiment results gained from the experiment setup of Figure 6(a) and (b), and their corresponding simulations. Figure 6(a)–(f) exhibit the simulation and experiment results of the structured lights mainly consisting of $V_{-31}$ and $V_{11}$ , $V_{21}$ and $V_{11}$ , $V_{31}$ and $V_{11}$ , $V_{21}$ and $V_{01}$ , $V_{-21}$ and $V_{11}$ vortex lights. Because the fields from Figure 6(a) may couple with other intrinsic modes when propagating in fiber, some other orders vortex modes besides the desired two orders may appear either. As a result, the intensity patterns seem to be messier than the fields from the SLM system. For the fields from the fiber system Figure 6(a), it’s troublesome to measure the concrete intensity and phase of all components. Therefore, a simulation is provided based on the results in the setup of Figure 6(a) to extract the two main components based on the locations and orientations of singularities, where the simulation intensity ratio is 90:10, 75:25, 80:20, 95:5, and 55:45. Meanwhile, the intensity ratio for the field from Figure 6(b) is measured to be 89:11, 78:22, 87:13, 95:5, and 57:43. It should be mentioned that the results gained from Figure 6(a) and (b) may exist inverse orders. For example, in Figure 7(c), the combination lights are $V_{21}$ and $V_{11}$ for the field from Figure 6(a) while $V_{-21}$ and $V_{-11}$ for the field from Figure 6(b). It doesn’t matter to the discussion of the singularities and interference resolution, but just with an inverse spiral or fork direction.

As can be seen, spiral interference patterns possess a relatively thick stripe, hard to distinguish the singularities in complicated modal combinations. Instead, when imposing a little tilt degree, the fork wire patterns may recognize these splitting singularities, even the intensity ratio up to 95:5.

**Figure 8:**
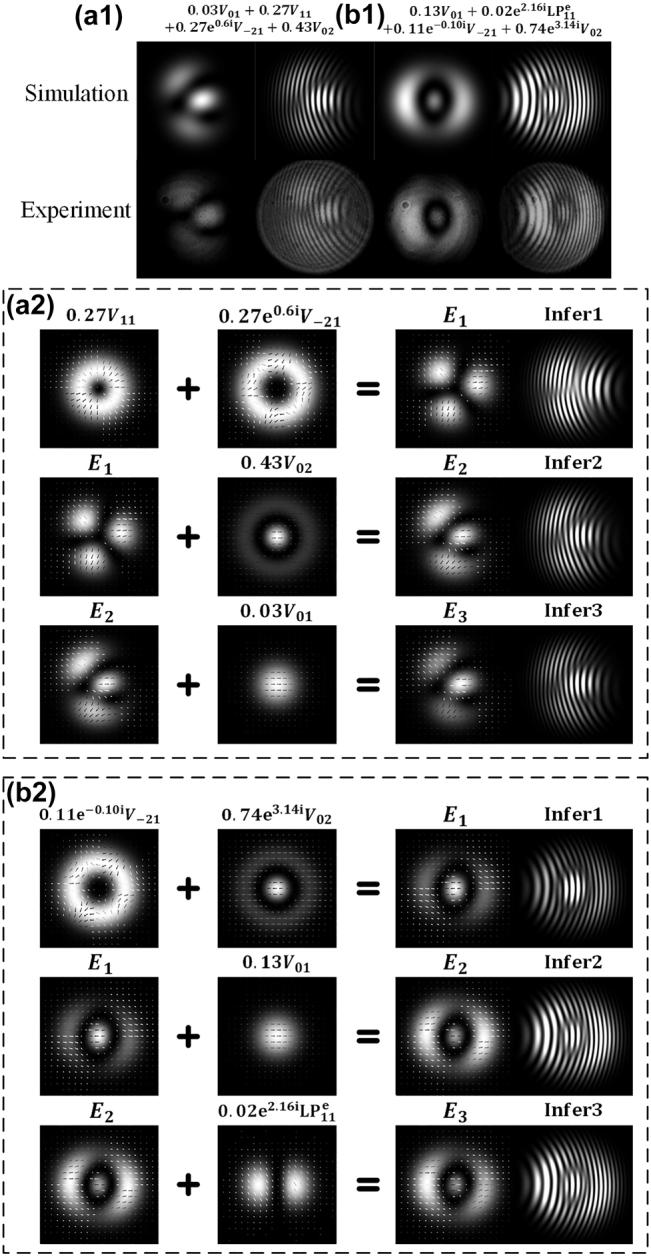
Process to recover detailed modal components based on the information of singularities obtained from detected light spots. The simulation and experiment patterns of structured lights from four-mode fiber with the recovered modal components as (a1) $0.03V_{01}+0.27V_{11}+0.27{\text{e}}^{0.6\text{i}}V_{-21}+0.43V_{02}$ and (b1) $0.13V_{01}+0.02{\text{e}}^{2.16\text{i}}{\text{LP}}_{11}^{\text{e}}+0.11{\text{e}}^{-0.10\text{i}}V_{-21}+0.74{\text{e}}^{3.14\text{i}}V_{02}$ , and the corresponding recovering processes are provided in (a2) and (b2). Infer, interference pattern.

Further, we’ll introduce how to gradually recover the detailed modal components based on the information of singularities from interference patterns. The fields shown in Figure 8 are generated in a four-mode fiber system through a fiber grating (−13 dB at 1545.7 nm for $V_{02}$ ). The interference patterns in Figure 8 are with some overexposure treatment to make the dark areas around singularities obvious. In Figure 8(a1), three singularities are revealed by fork wire interference pattern, where a lower opening fork wire on the left and two upper opening fork wires on the right. As the interference condition of Figure 8(a1) is that the reference fundamental light is left tilt and divergent related to the signal light, the upper opening fork wire represents a negative singularity while the lower opening fork wire represents a positive singularity, as the discussion in Figure 2. Unlike the field consisting of two vortex lights, there is not a clear central singularity in the results in Figure 8(a1). As a result, the field may possess more than two components with close intensity. It is needed to use the further conclusion of the field combined by two vortex lights, that is, regarding the hybrid field as the fundamental element and finding the effect of other modes on it. It’s reminiscent of the modal combination $E_{1}=V_{11}+V_{-21}$ , which possesses a positive original singularity and three negative non-origin singularities, as shown in the first row of Figure 8(a2). As mentioned in Figure 4(f), an additional phase uniform area may translate an existing singularity to the direction opposite to the phase difference of the area and signal light. Therefore, consider the effect of $V_{02}$ or $V_{01}$ , they both have the phase uniform area. Reconsider the location of three singularities in Figure 8(a1), the singularities have a left translation relative to the field $E_{1}$ , and the leftmost singularity is translated to the dark area and finally disappears. As the color of left arrows of the leftmost singularity of $E_{1}$ is red, we try to provide blue arrows to destructive interfere with these arrows so that the leftmost destructive point is translated toward the left. Exactly, $V_{02}$ with zero phase satisfy this condition, where the intensity of $V_{02}$ determines the translated length. Meanwhile, the other singularities are moved well. It will lead to the same result if one chooses another singularity to repeat the analysis process. Till this step, the simulation is quite close to the experiment. The rest component $V_{01}$ affects the intensity ratio of the central petal and the two next to it. Because the central petal is brighter than the other two, the arrows of $V_{01}$ should point to the right.

The analysis process is similar to the results in Figure 8(b1), where the interference condition is right tilt and divergent. Under the interference condition, the fork direction represents the inverse polarity compared with that in Figure 8(a1). Whatever, it’s not hard to recognize that the field mainly consists of $V_{02}$ and $V_{21}$ according to the distribution of singularities. The modal combination of $V_{02}$ and $V_{21}$ has been discussed in Figures 3(d) and 5(d). Then, the $V_{01}$ causes the destructive interference of the central area so that shrinks the central circle, shown in graphs of the second row in Figure 8(b2), and the ${\text{LP}}_{11}^{\text{e}}$ leads to the intensity asymmetry of the left petal and the right petal, shown in graphs of the third row in Figure 8(b2).

Based on the locations and orientations of singularities, we may have such a meticulous analysis to recover the amplitudes and phases of all modal components. However, it may take a little time to obtain all the amplitudes and phases in a high precision. For the experiment based on a fiber system, we may observe lots of modal combinations when disturbing fiber through the polarization controller or other devices. It is easy to estimate the main modal components by observing the locations and orientations of the singularities at the first sight, as has been done in Figure 7. The singularity analysis method may help researchers to rapidly estimate modal components and select their desired results to have further studies.

Meanwhile, this method may provide auxiliary discrimination of the generated structured lights in a fiber system. Take the structured light generated by this fiber grating for an example, even the peak at 1545.7 nm reaches −13 dB measured by the spectrometer, the final recovered $V_{02}$ component by the singularity analysis method is much less than 90%. Similar contradictions happen in the recovered modal components by this method and the declared conversion efficiency in previously reported articles. The explanation is that the lost energy of the fundamental mode detected by the spectrometer is not totally conversed to the desired mode. Based on this fact, it’s necessary to survey the optical fields in fiber from multiple perspectives.

**Figure 9:**
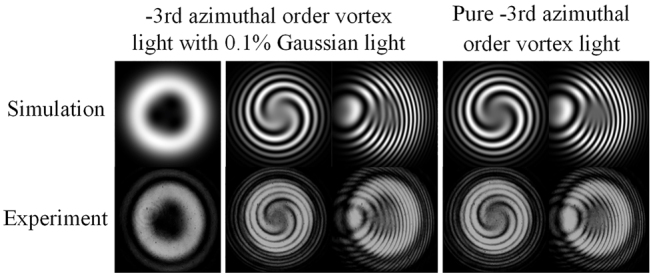
Intensity patterns and interference patterns for the structured light consisting of a −3rd azimuthal order vortex light and a Gaussian light under the intensity ratio of 1000:1 and a pure −3rd azimuthal order vortex light.

To further find the limited resolution of the fork wire pattern, an additional experiment under the SLM system is provided. The simulation and experiment results of structured light consisting of a −3rd and a 0th (Gaussian light) azimuthal order vortex light with the intensity ratio of 1000:1 is listed in the first to third columns of Figure 9. The singularities splitting phenomenon can still be observed in fork wire interference pattern, while the spiral interference pattern gains no information about that. As a comparison, the spiral and fork wire interference patterns of a pure −3rd azimuthal order vortex light are listed in the last two columns, where the three singularities concentrate at origin and form a triple degenerate singularity. Only in this case, the two kinds of interference patterns perform the same.

In theory, the interference resolution can be increased till the two interfering beams reach coherent length. However, the resolution is not able to be increased unboundedly in practice because of the limited resolution of the camera and the aperture of the optical path. Nevertheless, the fork wire pattern can still handle most of the impure cases even the intensity ratio up to 1000:1.

## 4 Conclusions

Singularities splitting phenomenon usually appears on the structured light consisting of hybrid orders vortex lights. Unlike pure vortex beams whose singularities concentrate at the center, hybrid-order vortex lights possess several singularities located around the space. The distribution of these splitting singularities mainly depends on the orders, amplitude ratio, and phase difference of combined vortex lights. Besides, these singularities may exhibit different orientations, that is, some of them are clockwise and others are counterclockwise.

To distinguish these splitting singularities, a Gaussian beam with different divergence (corresponding to the spiral interference pattern) and tilted angle (corresponding to the fork wire interference pattern) is usually used to interfere with the signal light. Because the singularities are close in space, the interference resolution becomes a vital evaluating indicator. Through discussion, the conventional spiral interference pattern is found to be the worst method to discriminate singularities because of its lowest resolution. Moreover, if insisting on using a spiral interference pattern, an inverse divergence between the signal beam and the reference beam is recommended to obtain higher resolution. However, in this interference condition, a positive singularity should correspond to the clockwise spiral pattern while a negative phase vortex corresponds to the counter-clockwise spiral pattern, which is contrary to mainstream judgment. On the other side, the fork wire interference pattern possesses the advantage of high resolution and easy adjustment. Furthermore, improving resolution by adjusting divergence may change the spot size, leading to the mismatch of the signal light and reference light. Instead, the spot size changes little when adjusting the tilted angle of the reference light. As a result, it is recommended to adjust the spot size of the reference light at first by changing the divergence (by adjusting the focus and location of the lens) to match the spot size of the signal light, and then change the tilted angle of the reference light to select the appropriate reference resolution.

Fork wire interference patterns are more appropriate to exhibit the experiment results instead of spiral interference patterns because they are with a higher interference resolution to discriminate the close singularities. By observing, the number, location, and orientation of splitting singularities, one may easily estimate the main modal components of the generated beam. Moreover, it’s possible to gradually recover the modal components. The analysis method is hoped to provide an extra perspective to research the fields emitted from few-mode fibers or multimode lasers.

## Supplementary Material

Supplementary Material
