# Supplementary material for: Singularities splitting phenomenon for the superposition of hybrid orders structured lights and the corresponding interference discrimination method
Source: Nanophotonics. 2022 Feb 24;11(7):1413–26. doi: 10.1515/nanoph-2021-0814 (PMC11501902; doi:10.1515/nanoph-2021-0814)
Supplement: Supplementary file 1 — Supplementary Material [file j_nanoph-2021-0814_suppl.docx]

Supplementary material for:

Singularities splitting phenomenon for the superposition of hybrid orders structured lights and the corresponding interference discrimination method

Baiwei Mao

In this material, we provide a further quantitative discussion about the resolution of spiral and fork-wire interference patterns.

Let’s have the detailed discussion of interference resolution from Eq.(2) in the manuscript,

$$E_{i}=A_{s}\left| E_{s}\left( r,\theta\right) \right|e^{i\gamma\left( r,\theta\right)}e^{ik_{z}z}+A_{f}e^{i\alpha_{f}}\cdot F_{01}\left( r \right)e^{i\left( k_{r}r^{2}+k_{x}x \right)}e^{ik_{z}z}$$

$$\begin{aligned} ={A_{s}^{2}\left| E_{s}\left( r,\theta\right) \right|}^{2}+\left[ A_{f}F_{01}\left( r \right) \right]^{2}+2A_{s}A_{f}\left| E_{s}\left( r,\theta\right) \right|F_{01}\left( r \right)\cos\left( \gamma\left( r,\theta\right)-k_{r}r^{2}-k_{x}x+\alpha_{l}-\alpha_{f} \right) \#\left( S1 \right) \end{aligned}.$$

When $k_{r}r^{2}+k_{x}x$changes 2$\pi$, A constructive line and a destructive line appear, which form a line pair. If a singularity locates in several line pairs, it can be recognized. The line pair density(line pair number per unit length) does not matter much when there is only a single singularity to be distinguished, as the pure second azimuthal order vortex light shown in Fig.S1(a). However, if several close singularities are to be distinguished, the line pair density will play an important role. As shown in Fig.S1(b), graphs of the second and the fourth columns are not able to distinguish the two close singularities under the low line pair density. As the comparison, graphs of the third and fifth columns can distinguish the close singularity because there is a stripe separating the fork wires or fork wire and vortex.


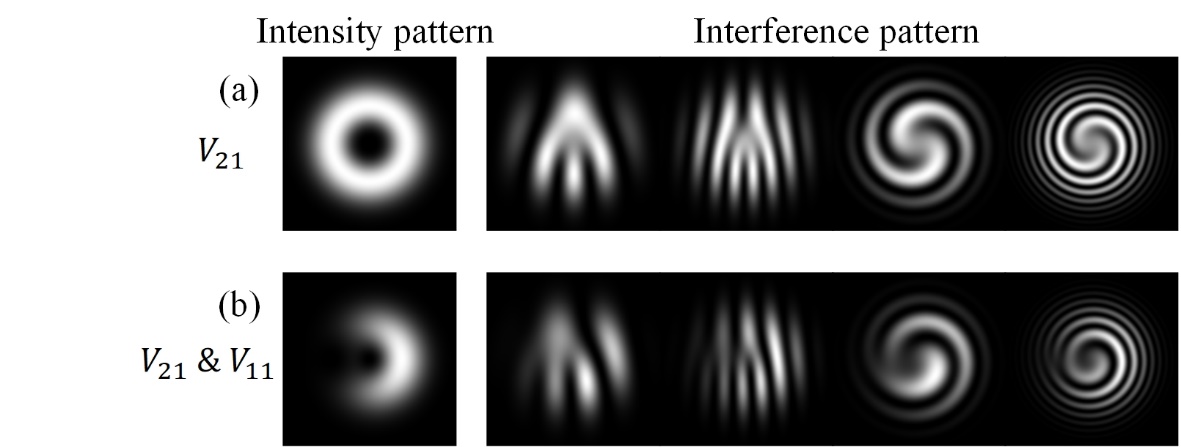


Fig.S1. Diagram to show the resolution of interference patterns under different line pair density, where the signal light is (a) a pure second-order vortex light $V_{21}$ and the combination of a second-order vortex light $V_{21}$ and a first-order vortex light $V_{11}$.

Line pair density is the quantization of the resolution of an interference pattern. To compare the superiority between spiral and fork wire patterns is equivalent to discussing the provided line pair density of a spherical wave$\left( k_{r}r^{2} \right)$ and an oblique plane wave$\left( k_{x}x \right)$. The line pair density can be easily derived as the derivates of $k_{r}r^{2}$ and $k_{x}x$ to their respective distance, that is $2k_{r}r$ and $k_{x}$. As indicated, the line pair density of the spiral pattern increases with the radius while that of the fork wire pattern is uniform.

There is another physical meaning of $2k_{r}r$ and $k_{x}$, as shown in Fig.S2. According to the definition of spatial frequency,

$$\begin{aligned} \left\{ \begin{aligned} \frac{\partial k_{r}r^{2}}{\partial r}=2k_{r}r=\sin\xi_{r} \\ \frac{\partial k_{x}x}{\partial x}=k_{x}x=\sin\xi_{x} \end{aligned} \right. \#\left( S2 \right) \end{aligned}.$$

where $\xi_{r}$ the divergent angle and $\xi_{x}$ is the oblique angle of the reference beam. The two beams fall on a camera with a diameter of $\left| \bar{\mathrm{AB}} \right|=2a$. Assume they commonly have $n$ line pairs on Line $\bar{\mathrm{AB}}$,

$$\begin{aligned} k_{r}a^{2}=k_{x}a=2n\pi\#\left( S3 \right) \end{aligned}.$$

Thus,

$$\begin{aligned} \frac{\sin\xi_{r}}{\sin\xi_{x}}=\frac{2k_{r}a}{k_{x}}=2 \#\left( S4 \right) \end{aligned},$$

which means $\xi_{r}>\xi_{x}$. The spiral interference pattern uses the line pair closest to the center to form the spiral pattern (if not using the central line pair, it should be a fork wire but not a spiral pattern). Using Eq.(S3), the span $d_{r}$ of the line pair for the spiral pattern should be

$$\begin{aligned} k_{r}d_{r}^{2}=\frac{2n\pi}{a^{2}}d_{r}^{2}=2\pi\to d_{r}=\frac{a}{\sqrt{n}} \#\left( S5 \right) \end{aligned}.$$

On the other hand, the span of arbitrary two line pairs is the same.

$$\begin{aligned} k_{x}d_{x}=\frac{2n}{a}d_{x}=2\pi\to d_{x}=\frac{a}{n} \#\left( S6 \right) \end{aligned}.$$

Therefore, under the condition Eq.(S3) and Eq.(S4), the line pair span of a tilted light is $\sqrt{n}$ smaller than that of a spherical light. As the number $n$ of line pair on $\bar{\mathrm{AB}}$ is a positive integer, $d_{x}\leq d_{r}$.

The above derivation is based on the condition $\sin\xi_{r}/\sin\xi_{x}=2$ so that $\xi_{r}>\xi_{x}$. For a larger $\xi_{x}$, the fork wire interference pattern performs better caused more dense line pairs can be generated. In reality, the divergent angle $\xi_{r}$ is probably much smaller than the oblique angle $\xi_{x}$. Because the propagating light usually satisfies the paraxial condition, the divergent angle of light is quite small(less than 0.01rad, $2k_{r}a=\sin\xi_{r}\approx0.01$). Assume a light with 1mm radius propagates 1m under the divergent angle of 0.01rad, the destined spot size is about 10mm. It’s a remarkable divergence and generally doesn’t occur. As a result, the real divergent angle should be smaller than 0.01rad. At the same time, the oblique angle $\xi_{x}$ may change from $-\pi/2$ to $\pi/2$ in theory. However, $\xi_{x}$ can not reach $\pm\pi/2$ because of the limited resolution of the camera and the aperture of the optical path. Even though, $\xi_{x}$ is much larger than $\xi_{r}$, lead to the conclusion that $k_{x}x$ changes much faster than $k_{r}r^{2}$. In short, the oblique of light can generate more dense line pairs than the divergence of light, so the tilted phase is more advantageous to distinguish close singularities in space.


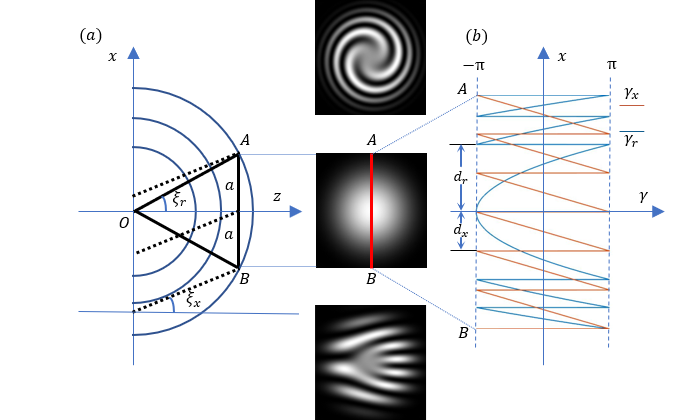


Fig.S2. Relation between the divergent angle $\xi_{r}$ and the oblique angle $\xi_{x}$ of the reference beam and the corresponding interference pattern. (a) The propagating process of a relative divergent(solid line) and a relative oblique reference light(dash line), which share the same image plane with the diameter $\left| \bar{\mathrm{AB}} \right|=2a$ and (b)the corresponding phase($\gamma$)-distance$\left( x \right)$ curve, where $d_{r}$ and $d_{x}$ are the span of the central line pair of spherical phase and tilted phase.
